# A scoping review and thematic analysis of social and behavioural research among HIV-serodiscordant couples in high-income settings

**DOI:** 10.1186/s12889-015-1488-9

**Published:** 2015-03-13

**Authors:** Joshua B Mendelsohn, Liviana Calzavara, Amrita Daftary, Sanjana Mitra, Joel Pidutti, Dan Allman, Adam Bourne, Mona Loutfy, Ted Myers

**Affiliations:** 1 grid.17063.33Dalla Lana School of Public Health, University of Toronto, Toronto, Canada; 2 grid.16463.360000000107234123Centre for the AIDS Programme of Research in South Africa (CAPRISA), University of KwaZulu Natal, Durban, South Africa; 3 grid.8991.9000000040425469XSigma Research Group, London School of Hygiene and Tropical Medicine, London, UK; 4 grid.17063.33Women’s College Research Institute, Women’s College Hospital, University of Toronto, Toronto, ON Canada

**Keywords:** High-income settings, HIV, AIDS, Serodiscordant, Relationships, Review, Social, Behavioural

## Abstract

**Background:**

While HIV incidence has stabilized in many settings, increases in health and wellbeing among many people living with HIV/AIDS suggest that the number of HIV-serodiscordant relationships is growing. Given the deficit of reviews addressing social and behavioural characteristics of HIV-serodiscordant couples within high-income settings, our objective was to understand the scope of the published literature, identify evidence gaps, and suggest future research needs.

**Methods:**

Ten electronic databases were searched. Studies were included if they were reported in English, used primary data, were from the combination antiretroviral (cART) era (>1996), reported on social or behavioural aspects, included any fraction of primary (i.e., stable) relationships, and were conducted in high-income settings. Studies that identified their unit of analysis as either the dyad or individual member of the couple were included. Studies were coded according to a thematic framework.

**Results:**

Included studies (n = 154) clustered into eight themes: risk behaviours (29%), risk management (26%), reproductive issues (12%), relationship quality (9%), serostatus disclosure (7%), adherence to antiretroviral therapy (7%), vulnerability (5%), and social support (3%). The proportion of studies conducted among heterosexual couples, same-sex male couples, and mixed cohorts were 42%, 34%, and 24%, respectively. Most studies (70%) were conducted in the United States, 70% of all studies were quantitative (including interventions), but only one-third were focused on couples (dyads) where both partners are recruited to a study. Over 25% of studies focused on sexual risk among same-sex male couples.

**Conclusions:**

Future research efforts should focus on the interrelationship of risk management strategies and relationship quality, social determinants of health and wellbeing, HIV testing, vulnerable populations, reproductive issues among same-sex couples, disclosure of serodiscordant status to social networks, dyadic studies, population-based studies, and interventions to support risk management within couples. Additional population-based studies and studies among marginalized groups would be helpful for targeting research and interventions to couples that are most in need. As HIV-positive partners are typically the link to services and research, innovative ways are needed for reaching out to HIV-negative partners. Our review suggests that significantly more research is needed to understand the social and behavioural contexts of HIV-serodiscordant relationships.

**Electronic supplementary material:**

The online version of this article (doi:10.1186/s12889-015-1488-9) contains supplementary material, which is available to authorized users.

## Background

Advances in HIV treatment and prevention have helped to stabilize HIV incidence in many countries. Concomitant increases in life expectancy and stabilized prevalence suggest that the number of primary HIV-serodiscordant couples (e.g., “serodiscordant couples” of mixed HIV status in which one partner is HIV-positive and the other is not, and who co-exist in a stable relationship) will increase [1,2]. Estimates suggest that in Sub-Saharan Africa, approximately half of HIV-positive persons in stable relationships have an HIV-negative partner [3]; however, there is an absence of similar data in high-income settings [4-6]. Given that regular sexual contact introduces a routine risk of HIV transmission, HIV-serodiscordant couples are a key population in which to understand how social and behavioural factors may affect risk perception and management, and inform comprehensive HIV prevention measures.

Although combination antiretroviral therapy (cART) has been shown to reduce the rate of new HIV infections within serodiscordant couples by 96% [7,8], the social and behavioural challenges that influence the successful scaling-up and integration of cART within serodiscordant relationships in non-trial, real-world settings are not fully understood. Pre-cART studies explored social and behavioural issues experienced by serodiscordant couples [9-14]; however, with advances in treatment, these issues are highly dynamic in the cART era. Behavioural interventions such as Couples HIV Testing and Counselling (CHTC) and HIV prevention educational interventions are not well understood among this key population. Their unique challenges include the management of sexual and emotional risk within the context of rapidly evolving treatment choices, illness-related stressors, HIV stigma within social networks, and a lack of supportive services, especially for the HIV-negative partner. In terms of levels of analysis, it is not clear whether most work in this area has primarily focused on the discordant dyad or individual partners within the couple and there have been no attempts to draw these different research approaches together into a single review.

Overall, there are no reviews that have synthesized knowledge in this area with the aim of identifying major gaps in evidence. Therefore, our main objective was to conduct a scoping review of the published literature in order to identify key evidence gaps and suggest future research needs in the field of HIV-serodiscordance.

## Methods

Scoping reviews are used to map a field of study and to determine the value in undertaking further systematic reviews or studies by helping to identify important evidence gaps [15]. For this review, PRISMA guidelines were adopted and used to help standardize reporting [16]. Ten electronic databases (Medline, EMBASE, CINAHL, Cochrane Collaboration, JSTOR, PsychInfo, Scopus, Web of Science, Sociological Abstracts, and Social Work Abstracts) were searched from November to December 2012. Search terms included “human immunodeficiency virus”, “HIV”, “acquired immune deficiency syndrome”, “AIDS”, “relation”, “relationships”, “couple”, “marriage”, “partner”, “spouse”, “serodiscordan*” and their equivalent Medical Subject Heading (MeSH) terms. These terms were combined using the generic formula: “HIV” and “serodiscordant” and “relationship”. For a sample search strategy, please see Additional file 1. No restrictions were initially applied with respect to geographic location, date, or language. After removing duplicates and mapping recurrent themes [17], we opted to focus the review by excluding citations that were not reported in English, did not report primary data, were conducted in the pre-cART era (≤1996), reported exclusively on casual or undefined relationships, did not report on social or behavioural aspects, or were conducted in low and middle-income settings [18]. We reviewed each study at the full-text review stage for evidence of a distinction between primary (i.e., stable) and casual relationships and included any study that incorporated any fraction of couples or individuals who were engaged in a primary relationship. Studies of social and behavioural aspects were included, while those covering clinical or biomedical topics (e.g., sperm washing), were excluded. Research on biomedical prophylaxes (e.g., treatment-as-prevention, TasP, or pre-exposure prophylaxis, PrEP) were included if the work focused on social or behavioural aspects. We opted to distinguish low- and middle-income settings from high-income settings and to focus the present review on the latter, given that disparate resource levels contribute to distinct socio-structural environments worthy of separate consideration. Studies that identified their unit of analysis as either the dyad (i.e., both members of the couple), as the individual member of the couple, or both, were included in this review.

One of two reviewers (SM or JP) made exclusion decisions. Citations for which exclusion criteria were inconclusive were noted and discussed. Where agreement could not be reached, the opinion of a third reviewer (JM) was solicited. The references cited list of all included articles identified within the original search of health science databases were checked for additional relevant citations. Following a framework analytic approach [19], we undertook a preliminary reading of included studies and developed an initial thematic framework based on the main outcomes reported. One researcher assigned each study to a primary theme within this framework. As coding progressed, new themes were incorporated into the framework and previously coded studies were re-assessed within the updated framework. Once a final framework was obtained, primary themes were assigned to each study by one researcher, and were validated by a second researcher. Discrepancies were discussed and resolved. Where appropriate, studies were assigned primary and secondary themes. For ease of presentation, results of included studies were grouped according to the primary theme. Each study was also categorized according to study location, methodology, population risk group, serostatus of included participants, and unit of analysis (i.e., individual or dyad) (see Table 1). This scoping review did not involve primary research with human subjects and therefore did not warrant institutional ethical approval.

**Table 1:** **Summary of included studies**

**1** ^**st**^ **author**	**Year**	**Ref #**	**Country**	**Type**	**N (n, SD)**	**Theme**								**Population**				**Unit of analysis**		**Serostatus**	
**✓**						**RM**	**RB**	**RQ**	**RI**	**SD**	**AD**	**SS**	**VU**	**MM**	**HE**	**WW**	**BI**	**IN**	**DY**	**+**	**-**
*Aidala*	2006	[31]	US	CO	278	✓(2)	✓(1)							✓	✓			✓		✓	
*Aoki*	2004	[115]	US	QL	1			✓(1)						✓				✓		✓	
*Asandar*	2009	[112]	SWD	QL	47					✓(1)		✓(2)			✓			✓		✓	
*Bairan*	2007	[86]	US	QL	104					✓(1)				✓	✓			✓		✓	
*Beckerman*	2000	[116]	US	CS	82			✓(1)					✓(2)	✓	✓				✓	✓	✓
*Beckerman*	2002	[117]	US	QL	24	✓(1)		✓(2)	✓(3)					✓	✓				✓	✓	✓
*Beckerman*	2002	[118]	US	MX	82			✓(1)					✓ (2)	✓	✓				✓	✓	✓
*Beckerman*	2002	[119]	US	QL	82			✓(1)	✓(3)				✓ (2)	✓	✓				✓	✓	✓
*Blashill*	2012	[39]	US	CS	430		✓(1)							✓				✓		✓	
*Bouhnik*	2007	[30]	FR	CS	663		✓(1)								✓			✓		✓	
*Bradley*	2008	[40]	US	CS	394		✓(1)							✓	✓				✓	✓	✓
*Brooks*	2011	[120]	US	QL	50	✓(1)	✓(2)							✓			✓		✓	✓	✓
*Brooks*	2012	[121]	US	QL	25	✓(1)	✓(2)							✓			✓	✓			✓
*Buchacz*	2001	[122]	US	CS	290	✓(1)	✓(2)								✓				✓	✓	✓
*Chakravarty*	2012	[59]	US	CS	752	✓(1)								✓				✓			✓
*Chen*	2001	[123]	US	CS	1421				✓(1)						✓	✓	✓	✓		✓	
*Chin*	1999	[87]	US	MX	9					✓(1)		✓(2)			✓			✓		✓	
*Coll*	1999	[124]	SP	CS	280		✓(2)		✓(1)						✓				✓	✓	✓
*Cooney*	2009	[125]	UK	CS	69				✓(1)						✓i			✓		✓	
*Corbett*	2009	[126]	US	QL	100	✓(1)	✓(2)								✓				✓	✓	✓
*Crane*	2002	[98]	US	QL	65								✓ (1)		✓				✓	✓	✓
*Cranson*	1998	[127]	US	QL	29		✓(2)	✓(1)						✓	✓	✓	✓	✓		✓	
*Crawford*	2001	[62]	AU	CS	1070	✓(1)	✓(2)							✓				✓		✓	✓
*Crawford*	2003	[37]	US	CS	230	✓(2)	✓(1)							✓			✓	✓		✓	✓
*Crawford*	2006	[128]	AU	CS	903	✓(2)	✓(1)							✓				✓		✓	✓
*Crosby*	2007	[129]	US	CS	1006		✓(1)						✓(2)	✓				✓		✓	✓
*Cusick*	1999	[84]	UK	QL	73					✓(1)				✓	✓		✓		✓	✓	✓
*Cusick*	2000	[64]	UK	QL	73	✓(1)	✓(2)							✓	✓		✓		✓	✓	✓
*Dave*	2006	[69]	UK	CS	142	✓(1)				✓(2)					✓			✓		✓	
*Davidovich*	2001	[130]	ND	CO	144		✓(1)							✓				✓		✓	
*Davis*	2011	[65]	UK	QL	16	✓(1)		✓(2)						✓				✓		✓	✓
*Dolezal*	2005	[131]	US	CS	234		✓(2)	✓(1)						✓					✓	✓	✓
*Doyal*	2005	[97]	UK	QL	62								✓(1)		✓			✓		✓	
*Eaton*	2008	[52]	US	CS	290	✓(1)							✓(2)		✓				✓	✓	✓
*Eaton*	2009	[132]	US	CS	254	✓(1)								✓					✓	✓	✓
*Eaton*	2009	[133]	US	CS	290		✓(1)								✓				✓	✓	✓
*El-Bassel*	2010	[99]	US	CS	1070 (535)		✓(2)						✓(1)		✓				✓	✓	✓
*El-Bassel*	2010	[21]	US	RCT	1070 (535)	✓(1)	✓(2)								✓				✓	✓	✓
*El-Bassel*	2010	[60]	US	CS	1070 (535)		✓(1)								✓				✓	✓	✓
*El-Bassel*	2010	[134]	US	CS	1070 (535)		✓(1)								✓				✓	✓	✓
*El-Bassel*	2010	[135]	US	CS	1070 (535)		✓(1)								✓				✓	✓	✓
*Elford*	1999	[56]	UK	CS	1004		✓(1)							✓				✓		NR	NR
*Elford*	2008	[111]	UK	CS	1687					✓(1)			✓(2)	✓	✓			✓		✓	
*Estes*	1997	[136]	US	CS	60			✓(1)				✓(2)		✓	✓	✓	✓				
*Fife*	2008	[28]	US	RCT	169			✓(2)			✓(3)	✓(1)		✓	✓	✓	✓		✓	✓	✓
*Fletcher*	2012	[72]	US	QL	42				✓(1)						✓			✓		✓	
*Fox*	2009	[61]	UK	CO	78	✓(2)	✓(1)							NR	NR	NR	NR		✓	✓	✓
*Galvan*	2004	[137]	US	CS	1421								✓(1)	✓	✓			✓		✓	
*Gaskins*	2009	[79]	US	CR	2								✓ (1)		✓			✓		✓	
*Gielen*	2000	[138]	US	MX	310					✓(2)			✓(1)		✓			✓		✓	
*Gordon-Garofa*	2004	[27]	US	QUA	29			✓(2)				✓(1)		✓	✓			✓			✓
*Gosselin*	2011	[71]	US	CS	286				✓(1)						✓				✓	✓	✓
*Greenberg*	2001	[88]	US	CS	238					✓(1)		✓(2)		✓					✓	✓	✓
*Guy*	2011	[139]	AU	CO	7857		✓(1)							✓				✓		✓	✓
*Guzman*	2006	[48]	US	CS	199	✓(1)	✓(2)							✓				✓		✓	✓
*Haas*	2002	[101]	US	QL	40			✓(2)				✓(1)		✓					✓	✓	✓
*Hart*	2005	[70]	US	CS	456	✓(1)				✓(2)				✓				✓		✓	
*Heard*	2004	[140]	FR	CO	575				✓(1)						✓ii			✓		✓	
*Heard*	2007	[141]	FR	CO	1254				✓(1)						✓			✓		✓	
*Hernando*	2009	[24]	SP	CO	1128	✓(1)	✓(2)								✓				✓	✓	✓
*Hoff*	2004	[36]	US	MX	245		✓(1)							✓				✓		✓	
*Hoff*	2009	[57]	US	CS	382 (90)	✓(1)								✓					✓	✓	✓
*Hoff*	2010	[63]	US	QL	78	✓(1)		✓(2)						✓					✓	✓	✓
*Horvath*	2012	[47]	US	CS	326	✓(1)	✓(2)							✓				✓		✓	✓
*Hotton*	2011	[142]	US	CS	793	✓(1)	✓(2)							✓				✓		✓	✓
*Hunt*	1999	[143]	US	CS	52		✓(1)						✓(1)		✓			✓		✓	✓
*Israel*	2005	[41]	US	CS	74		✓(1)							✓	✓	✓		✓			✓
*Jarman*	2005	[144]	UK	QL	6			✓(1)					✓(2)		✓	✓		✓		✓	
*Johnson*	2011	[90]	US	CS	420						✓(1)			✓			✓		✓	✓	✓
*Kalichman*	1999	[80]	US	CS	266		✓(2)			✓(1)				✓iii	✓			✓		✓	
*Kalichman*	1999	[145]	US	CS	331		✓(1)						✓(2)	✓iv	✓	✓	✓	✓		✓	
*Kalichman*	2002	[32]	US	CS	383		✓(1)							✓	✓		✓	✓		✓	
*Kelly*	2011	[74]	UK	QL	10	✓(3)	✓(2)		✓(1)						✓			✓		✓	
*Klein*	2003	[146]	US	MX	100				✓(1)						✓v				✓	✓	✓
*Knight*	2005	[147]	US	MX	161		✓(1)						✓(2)	✓	✓		✓	✓		✓	
*Knowlton*	2011	[92]	US	CS	145						✓(1)	✓(2)			✓vi			✓		✓	
*Knowlton*	2011	[94]	US	CS	104						✓(1)	✓(2)			✓vii			✓		✓	
*Latka*	2006	[44]	US	CS	426 (292)		✓(1)						✓(2)		✓			✓		✓	
*Liu*	2011	[68]	US	CO	2266	✓(1)	✓(2)								✓			✓		✓	✓
*Lopez*	2010	[148]	US	CO	190						✓(1)		✓(2)		✓			✓	✓	✓	
*Marks*	2001	[149]	US	CS	206		✓(2)			✓(1)				✓	✓		✓	✓		✓	
*Marujo*	2012	[150]	PO	CO	71				✓(1)						✓			✓		✓	
*McDonald*	2011	[151]	AU	QL	34		✓(2)		✓(1)						✓			✓		✓	
*McFarland*	2011	[152]	US	CS	1207	✓(1)	✓(2)							✓				✓		✓	✓
*McFarland*	2012	[153]	US	CO	732	✓(1)	✓(2)							✓				✓		✓	✓
*Milam*	2006	[29]	US	CS	121		✓(1)								✓			✓		✓	
*Mizuno*	2007	[154]	US	CS	348	✓(2)	✓(1)						✓(2)		✓			✓		✓	
*Myers*	2006	[100]	US	CS	147		✓(2)						✓(1)		NR			✓		✓	
*Nakhuda*	2005	[155]	US	CR	5				✓(1)						✓			✓		✓	
*Niccolai*	2002	[81]	US	CS	76			✓(2)		✓(1)				✓	✓	✓			✓	✓	✓
*Nichols*	2006	[156]	US	CS	150			✓(1)					✓(2)	✓					✓	✓	✓
*NIMH*	2008	[20]	US	MX	86	✓(2)	✓(1)								✓				✓	✓	✓
*Noestlinger*	2012	[73]	EU	CS	427				✓(1)						✓			✓		✓	
*Nostlinger*	2010	[157]	EU	CS	651	✓(1)	✓(2)								✓			✓		✓	
*Operario*	2011	[46]	US	CS	174		✓(1)							✓	✓			✓		✓	✓
*Operario*	2011	[45]	US	CS	174		✓(1)							✓	✓			✓		✓	✓
*Ostrow*	2002	[105]	US	CS	547	✓(2)	✓(1)							✓				✓		✓	✓
*Ostrow*	2008	[58]	US	CO	501	✓(2)	✓(1)							✓				✓		✓	✓
*Palmer*	2001	[78]	UK	QL	20	✓(2)		✓(1)					✓(3)	✓					✓	✓	✓
*Panozzo*	2003	[158]	SW	CS	114	✓(3)	✓(2)		✓(1)						✓			✓		✓	
*Parish*	2001	[159]	US	QL	79	✓(2)	✓(1)								✓			✓	✓	✓	✓
*Parsons*	2004	[82]	US	MX	158		✓(2)			✓(1)				✓	✓		✓	✓		✓	
*Parsons*	2005	[160]	US	CS	1168	✓(1)	✓(2)							✓			✓	✓		✓	
*Peretti-Watel*	2006	[96]	FR	CS	1809		✓(2)				✓(1)		✓(3)	✓	✓		✓	✓		✓	
*Persson*	2011	[77]	AU	QL	14			✓(2)					✓(1)		✓			✓			✓
*Persson*	2008	[76]	AU	QL	46			✓(1)							✓			✓	✓	✓	✓
*Persson*	2008	[161]	AU	QL	16	✓(3)	✓(2)						✓(1)		✓			✓	✓	✓	✓
*Persson*	2010	[54]	AU	QL	27	✓(2)	✓(1)	✓(3)							✓			✓	✓	✓	✓
*Poindexter*	2003	[162]	US	QL	2			✓(1)					✓(2)		✓				✓	✓	✓
*Pomeroy*	2002	[25]	US	QUA	24			✓(1)							✓				✓	✓	✓
*Poppen*	2005	[34]	US	CS	219	✓(3)	✓(1)			✓(2)				✓				✓		✓	
*Prestage*	2008	[163]	AU	CS	23424 (2489)	✓(1)								✓				✓		✓	✓
*Prestage*	2009	[49]	AU	CO	1006	✓(1)	✓(2)							✓				✓		✓	✓
*Reilly*	2001	[164]	US	CS	360		✓(1)							✓	✓	✓	✓	✓		✓	
*Reilly*	2004	[165]	US	CS	360		✓(2)					✓(1)		✓	✓	✓	✓	✓		✓	
*Remien*	2005	[26]	US	RCT	430						✓(1)			✓	✓	✓		✓	✓	✓	✓
*Rhodes*	2000	[66]	UK	QL	73	✓(1)		✓(2)						✓	✓		✓	✓	✓	✓	✓
*Ritchie*	2012	[166]	UK	CO	275 (192)		✓(1)							✓	✓				✓	✓	✓
*Ross*	2008	[167]	US	CS	675		✓(1)			✓(3)			✓(2)	✓				✓		✓	
*Rosser*	2010	[22]	US	RCT	675	✓(1)	✓(2)							✓				✓		✓	
*Schonnesson*	2008	[168]	US	CS	258		✓(1)								✓			✓		✓	
*Semple*	2002	[169]	US	CS	47		✓(1)								✓			✓		✓	
*Semple*	2006	[35]	US	CS	132		✓(1)			✓(2)				✓				✓		✓	
*Service*	2006	[170]	US	CS	604 (172)	✓(2)	✓(1)							✓	✓		✓	✓		✓	
*Sherr*	2004	[171]	UK	CS	32				✓(1)						✓			✓		✓	
*Simoni*	2000	[42]	US	CS	105		✓(1)	✓(2)							✓			✓		✓	
*Sowell*	1999	[172]	US	CS	45				✓(1)						✓			✓		✓	
*Sowell*	2003	[85]	US	MX	322					✓(1)					✓			✓		✓	
*Stevens*	2007	[173]	US	QL	55		✓(1)						✓(2)		✓			✓		✓	
*Stirratt*	2006	[95]	US	CS	215					✓(2)	✓(1)			✓	✓	✓	✓	✓		✓	
*Stolte*	2004	[53]	ND	CO	57	✓(1)	✓(2)							✓				✓		✓	
*Stumbo*	2011	[174]	US	QL	40						✓(1)	✓(2)		✓					✓	✓	✓
*Sullivan*	2009	[83]	US	CS	99		✓(2)			✓(1)				✓	✓		✓	✓		✓	
*Sunderam*	2008	[75]	IT	QL	33				✓(1)						✓			✓	✓	✓	✓
*Suzan-Monti*	2011	[175]	FR	CS	322	✓(1)								✓				✓		✓	
*Theall*	2007	[43]	US	CS	187		✓(1)				✓(2)				✓viii			✓		✓	
*Theodore*	2004	[38]	US	CS	87		✓(1)	✓(2)						✓				✓		✓	
*Tieu*	2011	[33]	US	CS	328		✓(1)			✓(2)				✓				✓		✓	✓
*Turner*	1998	[176]	US	CS	642							✓(1)		✓ix	✓		✓	✓			✓
*Van de Ven*	2002	[177]	AU	CS	14156	✓(1)	✓(2)							✓				✓		✓	✓
*Van de Ven*	2005	[50]	AU	CS	119	✓(1)	✓(2)							✓				✓		✓	✓
*Van der Straten*	2000	[51]	US	CS	208	✓(1)	✓(2)								✓				✓	✓	✓
*Van Leeuwen*	2008	[178]	ND	CS	100				✓(1)						✓				✓	✓	✓
*Vide Tavares*	2012	[179]	PO	CS	133				✓(1)						✓x			✓		✓	
*Wagner*	2002	[89]	US	CS	80						✓(1)			✓	✓				✓	✓	✓
*Weinhardt*	2004	[180]	US	CS	3723		✓(1)							✓	✓	✓	✓	✓		✓	✓
*Wilson*	2007	[104]	US	CO	1090	✓(1)	✓(2)								✓			✓		✓	
*Wolitski*	2003	[181]	US	CS	250	✓(1)	✓(2)							✓				✓		✓	
*Wrubel*	2008	[93]	US	QL	40						✓(1)			✓					✓	✓	✓
*Wrubel*	2010	[91]	US	QL	40						✓(1)	✓(2)		✓					✓	✓	✓
*Wyatt*	2004	[23]	US	RCT	147	✓(1)	✓(2)				✓(3)		✓(4)		✓xi			✓		✓	
*Wyatt*	2012	[182]	US	CR	2	✓(1)	✓(2)								✓				✓	✓	✓
*Xia*	2006	[67]	US	CS	354	✓(1)	✓(2)							✓				✓		✓	✓
*Zablotska*	2011	[183]	AU	CO	16022	✓(1)	✓(2)							✓				✓		✓	✓

i Population not explicitly stated; assumed heterosexual because sample of women address fertility needs.

ii Population not explicitly stated; assumed heterosexual because a majority of women contracted HIV through heterosexual contact.

iii Population not explicitly stated; assumed men who have sex with men, and heterosexual because of sexual acts reported.

iv Population not explicitly stated; assumed men who have sex with men, heterosexual, women who have sex with women and bisexual because of partners reported.

v Population not explicitly stated; assumed heterosexual because sample included men and women in couples with whom fertility concerns were explored.

vi Population not explicitly stated; assumed heterosexual because a majority of men contracted HIV through heterosexual contact.

vii Population not explicitly stated; assumed heterosexual because a majority of women contracted HIV through heterosexual contact.

viii Population not explicitly stated; assumed heterosexual because a majority of women contracted HIV through heterosexual contact.

ix Population not explicitly stated; assumed men who have sex with men, heterosexual and bisexual because of sample characteristics.

x Population not explicitly stated; assumed heterosexual because the sample of women addressed contraception use.

xi Population not explicitly stated; assumed heterosexual because of sexual acts reported.

Legend. LOCATION: United States (US); United Kingdom (UK); Australia (AU); Multiple European Union Countries (EU); France (FR); Italy (IT); Netherlands (ND); Spain (SP); Sweden (SWD); Switzerland (SW); Portugal (PO). TYPE: cross-sectional study (CS); case control (CC); case report (CR); cohort study (CO); quasi-experimental (QUA) randomized controlled trial (RCT); qualitative study (QL); mixed methods (MX). THEME: risk management (RM); sexual risk behaviours (RB); relationship quality (RQ); reproductive issues (RI); serostatus disclosure (SD); adherence (AD); social support (SS); vulnerability (VU). POPULATION: men who have sex with men (MM); heterosexual (HE); women who have sex with women (WW); bisexual (BI). UNIT OF ANALYSIS: individual (IN); dyad (DY). SEROSTATUS: HIV-positive (+); HIV-negative (−). SAMPLE SIZE: no. serodiscordant (n,SD). Data not reported (NR).

## Results

**Figure 1:**
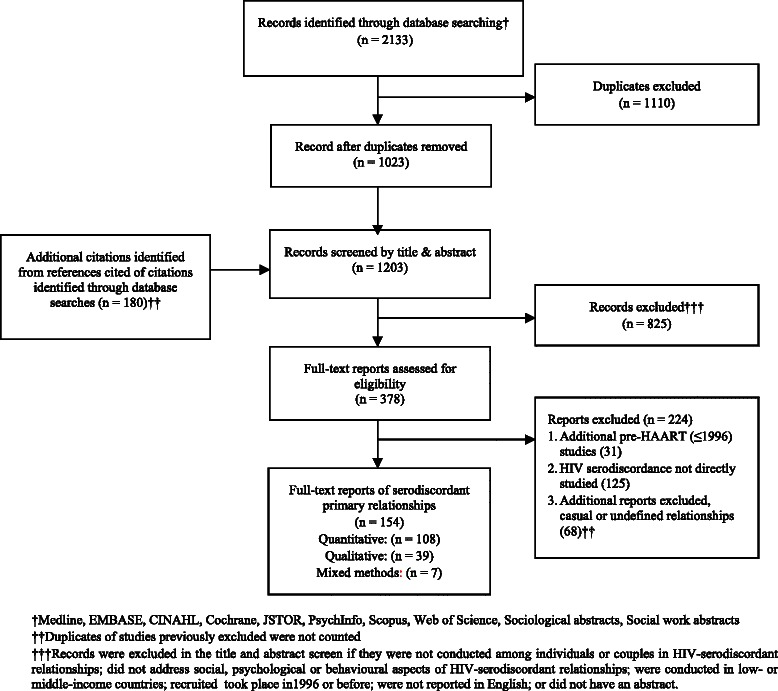
**Study selection flowchart.**

**Figure 2:**
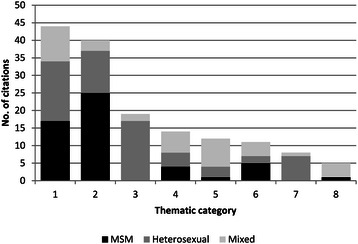
**Citations stratified by risk group and thematic area (1 = risk behaviours; 2 = risk management; 3 = reproductive issues; 4 = relationship quality; 5 = serostatus disclosure; 6 = adherence; 7 = vulnerability; 8 = social support).**

Searches yielded a total of 2,133 citations, of which 154 were included in this review (Figure 1). These citations clustered according to eight primary themes: sexual risk behaviours (29%; 45/154), risk management (26%; 40/154), reproductive issues (12%; 19/154), relationship quality (9%; 14/154), serostatus disclosure (7%; 12/154), adherence to cART (7%; 11/154), vulnerability (5%; 8/154), and social support (3%; 5/154). The proportions of included studies that were conducted among heterosexual serodiscordant couples, same-sex male couples, and mixed cohorts were 42% (64/154), 34% (53/154), and 24% (37/154), respectively. Over 70% (109/154) were conducted within the United States, 21% (32/154) were European, and 8% (13/154) were Australian. The focus of over one-quarter of identified studies was sexual risk behaviours among same-sex male couples (Figure 2). Methodologically, 70% (108/154) of studies used quantitative methods (6%; 9/154 were intervention studies), 25% (39/154) used qualitative methods, and 5% (7/154) were mixed methods. Half of qualitative studies (21/39) were conducted among individuals or couples within heterosexual serodiscordant relationships. One-third (53/154) of studies used a dyadic unit of analysis, meaning that both partners participated in the study. Intervention studies (counted as quantitative studies) included behavioural interventions to reduce sexual risk behaviours [20-24], a small psycho-education group intervention to reduce depression and anxiety and increase marital satisfaction [25], an intervention to improve adherence to cART [26], and two interventions for improving social support [27,28].

### Sexual risk behaviours

The “sexual risk behaviour” theme was assigned to any study that assessed the prevalence of, or risk factors associated with, sexual risk behaviours. Relationships of longer duration were typically associated with more sexual risk, while evidence of the association between risky sexual behaviours and relationship type was mixed. Among a cohort of heterosexual partners recruited in California, longer relationship duration was associated with unprotected vaginal intercourse (UVI) but not relationship type (e.g., primary or casual) [29]. In a French study conducted among HIV-positive heterosexual individuals engaged in regular serodiscordant relationships or relationships of unknown status, sexual risk was linked to relationships of >10 years duration, financial difficulties, and routine excessive alcohol consumption among men [30]. Among HIV-positive heterosexual American men, UVI was associated with being in a primary partnership, age, sex with men, and exchanging sex for money or drugs [31]. In a comparison of American men and women with casual and regular serodiscordant partners, there was a fourfold increase in the risk that regular partners had UVI [32]. Among African-American HIV-negative men who have sex with men (MSM), being in a non-primary partnership was the only factor associated with serodiscordant or serostatus unknown unprotected anal intercourse (UAI) [33]. However, among Latino MSM, serodiscordance, serostatus disclosure, and relationship type (e.g., primary, casual) were not associated with sexual risk [34]. HIV-positive methamphetamine-using MSM engaged in risky sex with a range of partner types including steady, casual and anonymous partners, but less UAI was reported among those in serodiscordant relationships [35].

In some studies, couples appeared to manage and reduce risky sex when compared with other groups. For example, American MSM in primary seroconcordant relationships reported significantly more UAI and were more likely to have sex outside of their relationships when compared with those in primary serodiscordant relationships; yet, the impact of the level of intimacy on risky sex was moderated by drug use before sex and the partners’ HIV status [36-38]. In studies of mental health, depressed HIV-positive partners were less likely to report unprotected sex but more likely to have outside partners [39,40]. Among American HIV-negative partners in serodiscordant relationships, “sensation-seeking” increased risky sex by 3–5 times [41].

Among women, those who were HIV-positive and in primary partnerships were more likely to have unprotected sex than HIV-positive women without partners in the US [42]. Women who binged on alcohol or were on cART were also more prone to risky sexual behaviour [43]. Among female IDUs, inconsistent condom use was more likely to occur in relationships where mutual disclosure of HIV status had not occurred [44]. Among transgender women, risk behaviours were not associated with type or duration of relationship; however, living with a primary partner, drug and alcohol use, education level, low condom self-efficacy, and perceived discrimination were associated with unprotected sex among primary serodiscordant couples [45]. Among men with a primary discordant transgender partner, risk factors for unprotected sex were younger age, concurrent partnerships, alcohol intoxication, and low condom use self-efficacy [46].

### Risk management

The “risk management” theme was assigned to studies that focused on efforts to avoid or manage the risk of infection and included work on condom use, PrEP, viral load knowledge, negotiated safety agreements, seroadaptation (e.g., “seropositioning” or selection of partners according to serostatus and “serosorting” or strategic positioning during sexual intercourse), HIV testing, and serostatus disclosure.

Given that it is now accepted that an undetectable viral load reduces the risk of infection, investigations into the effects of actual and perceived viral load are of particular interest. An online survey that studied the impact of viral load discussions on risky sex among American MSM found that UAI occurred within 25% of primary serodiscordant couples who had discussed their viral load together [47]. A study in San Francisco found that viral load discussions were not more common among men in serodiscodant primary relationships compared with men who were not in such relationships; however, HIV-negative men who typically discussed viral load reported that they would be more inclined to take sexual risks with their HIV-positive partners whose viral load was undetectable [48]. In Australian studies, having an undetectable viral load was associated with a threefold increase in the odds of UAI among same-sex male serodiscordant couples and was more likely to be reported by seronegative partners when they believed their HIV-positive partner’s viral load was undetectable [49,50]. In the *California Partners II* study, HIV-negative partners estimated a lower likelihood of HIV transmission and had riskier sex if they thought that their partner was on cART and had an undetectable viral load [51,52]. Favourable perceptions of viral load among HIV-positive MSM based in Amsterdam led to increases in risk behaviours with seronegative or unknown status partners when compared with men who had an unfavorable perception of their viral load, even after adjusting for actual viral load and CD4 counts [53]. In an Australian qualitative study, perceptions of viral load, infectiousness, and safety of unprotected sex were linked to emotional sensibilities and commitments, as much as risk calculations [54].

Negotiated safety agreements (i.e., the negotiation of mutually agreed strategies for managing HIV risk within sexual relationships) and seroadaptation were the most commonly discussed behavioural risk management strategies within the surveyed literature [55]. Between 26% and 91% of cohorts included in this review reported having used sexual agreements [56-59]. Among African-American serodiscordant couples, “couple agreement” in reporting of sexual risk behaviours was consistent [60]; however, individual partners were found to hold different perceptions of transmission risk [61]. Among same-sex male couples based in Australia, sexual agreements that included casual serodiscordant partners, or having no agreement, were associated with UAI [62]. Qualitative work conducted in San Francisco found very little uniformity in the rules of negotiated agreements across couples [63]. In a British study, HIV-negative partners in both heterosexual and same-sex primary serodiscordant couples often requested risky sex in order to demonstrate love and relationship investment, or to reinforce trust within the relationship. This finding reinforces the importance of understanding the emotional and intimate dynamics of relationships when designing interventions [64,65]. In critiquing the focus on the individual costs and benefits of risk management, a British qualitative study noted that research on sexual risk decision-making often ignored the intimate and emotional experience of couples, the major context in which such decision-making is undertaken [66]. Seroadaptive behaviours include seropositioning (i.e., where seronegative men position themselves as the insertive partner) and serosorting (i.e., selection of partners on the basis of serostatus). Seropositioning was adopted by 58% of HIV-negative MSM in a population-based study in California [67]. Among women based in six US cities, both HIV-positive and seronegative female partners engaged in serosorting [68].

In studies that explored associations between risk management and HIV serostatus disclosure, knowledge of a heterosexual partner's HIV status was associated with condom use among those who had disclosed their serostatus [69]. Frequencies of risk behaviour were similar among MSM whether they were consistently aware or unaware of their partners’ HIV infection [70]. In the only study that examined HIV testing, 72% of seronegative men in same-sex serodiscordant couples who reported UAI with their HIV-positive primary partner or had UAI with an outside partner of discordant or unknown status had tested for HIV in the past year [59].

Four studies reported on interventions that aimed to reduce sexual risk behaviours. In a cluster randomized trial of a risk reduction intervention for African-American serodiscordant couples that addressed stigma and psychological distress, barriers to condom use, support from community organizations, and relationship skills, a lower adjusted mean number of unprotected sex acts was found in the intervention group [21]. In a non-randomized Spanish trial that evaluated the impact of a Couples’ Counselling and Testing intervention on sexual risk, pre-intervention sexual risk behaviour, >35 years of age, and a recent pregnancy increased the odds of post-intervention risky sexual behaviour by 2–3 times [24]. A randomized controlled trial of two sexual health seminars conducted among MSM found that UAI frequency decreased in all study conditions despite better reported intentions to avoid transmission among those in the intervention arms [22]. In a US-based psycho-educational intervention designed to decrease sexual risk and improve adherence to cART among heterosexual HIV-positive women with histories of child sexual abuse, the intervention was associated with a threefold increase in the odds of reducing sexual risk while those who attended ≥8 sessions reported a fourfold improvement in adherence compared to the control group [23]. Although there is some evidence that interventions have a positive impact on sexual risk behaviours among serodiscordant couples, overall there is a paucity of evidence from which to draw.

### Reproductive issues

Studies were assigned the “reproductive issues” theme if they focused primarily on any social or behavioural aspect of reproduction or fertility. Notably, studies classified within this theme were conducted exclusively among heterosexual serodiscordant couples. In one study of American couples’ desire to conceive through assisted reproduction where the majority of men in the study had previously been diagnosed with HIV, participants were most concerned about the risk of transmission within the family, premature death of the HIV-positive partner, disclosure of HIV status to children, and the potential desire for additional children if assisted reproduction proved successful on the first attempt [71]. In a qualitative study, 93% of participating women in South Carolina had no desire to become pregnant due to concerns about HIV transmission to the fetus, financial instability, single relationship status, age, and previous childbearing or child rearing experiences [72]. A European study that explored satisfaction with sexual and reproductive health services reported that 58% of all participants (59% of sample were in a serodiscordant couple) were not satisfied with services delivered in HIV care settings, while a third of participants reported HIV-related discrimination in healthcare settings [73]. Qualitative work conducted in Northern Ireland found that love, commitment, physical pleasure, and a desire to conceive without medical interventions helped to shape perceptions of risk within the relationship [74]. Among Italian couples consisting of HIV-positive men and seronegative women, assisted reproduction was typically regarded as safe and effective [75].

### Relationship quality

Studies were assigned the “relationship quality” theme if they explored relationship satisfaction or outcomes of serodiscordant relationships. Two-thirds (11/18) of these studies used qualitative methods. The “Straightpoz” study, an Australian longitudinal cohort of serodiscordant couples, found that HIV-positive partners felt that their identities were “redeemed” by their seronegative partners and revealed tensions within the couple surrounding communication about serodiscordance [76,77]. Among gay male serodiscordant couples, relationship balance and redefinition of the sexual relationship resulted when serodiscordance was uncovered by the couple after the relationship had started [78]. A pilot study testing a psycho-educational group intervention for heterosexual serodiscordant couples found reductions in depression and anxiety and increases in marital satisfaction among the intervention group [25].

### Serostatus disclosure

Studies were assigned to the “serostatus disclosure” theme if the primary finding was related to disclosure of serostatus to partners, family, or wider social networks. Serostatus disclosure, however, typically referred to individual disclosure of HIV status to partners. The finding that 41% and 47% of HIV-positive men and women engaged in a serodiscordant couple, respectively, had not disclosed their status to their partner [79,80] was consistent with disclosure rates found in other studies [81-83]. Qualitative work conducted in the UK suggested that negative ideas about condoms, concerns about relationship quality, and limited communication within the relationship, were barriers to disclosing serostatus to partners [84]. In a mixed-methods study conducted among American women, qualitative interviews revealed that disclosure decisions for women were often based on emotional closeness to their partner, or were organized around a highly personalized (i.e., non-generalizable) set of criteria [85]. In a study conducted among American HIV-positive IDUs, there was a higher likelihood of disclosure to casual partners before first sexual contact when compared to primary partners [82]. Disclosure of HIV status to social networks appeared to present a different type of issue upon which very few studies have focused. A qualitative study conducted among MSM, high-risk heterosexuals, and substance users found that the type of social relationship (e.g., sexual vs non-sexual; close vs not close) was a key consideration within the disclosure decision process [86]. Among Asian/Pacific Islanders, fears of stigma or discrimination, and concerns about disappointing or burdening others, were important to the process of disclosing HIV status to social networks [87]. HIV-positive partners were more likely to disclose their serodiscordant relationship to social networks when compared with their seronegative partners as a result of better self-reported social integration [88].

### Adherence to antiretroviral therapy

Studies were classified within the “adherence” theme if their main finding was related to cART adherence. Studies found that strong social support was linked to better adherence by the HIV-positive partner within serodiscordant couples. In a pilot-study of 40 couples based in the United States, higher adherence levels were associated with less risk attribution to unprotected sex by seronegative partners, better perceived treatment efficacy, good knowledge of treatment, and the perceived negative consequences of poor adherence [89]. Highlighting the importance of dyadic approaches to the study of adherence within couples, the seronegative partner’s estimate of adherence was found to be a better predictor of viral suppression than the HIV-positive partner’s own self-reported adherence in a cohort of same-sex male couples [90,91]. Among men, adherence was strongly associated with comfort in taking cART in the presence of close friends and support from caregivers [92,93]. Among American women, good adherence was 75% less likely among seroconcordant couples when compared to serodiscordant couples, and 78% less likely in situations where the HIV-positive partner was dependent on emotional support from their partner [94]. In a New York study that examined the relationship between HIV serostatus disclosure and adherence, disclosure was independently associated with better adherence [95]. In a French study that examined the co-occurrence of non-adherence to cART and risky sex within serodiscordant couples, no differences were observed in the proportions engaged in unsafe sex by adherence level (high vs. non-) among MSM and heterosexual men; however, a positive association was found among heterosexual women [96]. An educational and counseling intervention to improve adherence among heterosexual and same-sex serodiscordant couples in New York found short-term improvements in the proportion of prescribed doses taken correctly by the HIV-positive partner within the intervention group [26].

### Vulnerability and social support

The “vulnerability” theme was assigned when the primary contribution was a focus on understudied or highly marginalized groups such as migrants, those living in poverty, or those who had suffered from abuse. Although IDUs are highly vulnerable, these studies were typically assigned to other themes, and secondarily coded within the vulnerability theme. Among immigrant women from sub-Saharan Africa who were receiving HIV treatment in the United Kingdom, vulnerability was described by women as an interplay between migration, HIV status, and poverty [97]. A qualitative study of 33 low-income, marginally-housed, or substance using heterosexual serodiscordant couples situated in California found that HIV diagnosis was typically used as a commodity that allowed individuals to retain their access to crucial government programs that had been scaled back as a consequence of welfare reforms [98]. In a study of adult and child sexual abuse among African American serodiscordant couples, 72% of couples reported a history of abuse among one or both partners [99]. Child sexual abuse and adult re-victimization were independent risk factors for post-traumatic stress disorder and sexual trauma symptoms, but were not associated with risky sexual behaviour among HIV-positive women [100].

In qualitative studies of social support, partners were the primary source of support among same-sex male serodiscordant couples, while less support was received from families [101]. In a small quasi-experimental study that evaluated the effect of a psychoeducational group intervention on within-couple support, the intervention did not increase perceived social support [27]. Moreover, an intervention that attempted to improve coping and social support within couples led to positive changes in active coping, positive refocusing and less emotional distress, but did not result in a reported improvement in social support [28]. Therefore, evidence-based, couple-focused interventions that aim to increase supportive relationships among serodiscordant couples are currently lacking.

## Discussion

Through the process of reviewing the 154 included citations and assigning them to eight thematic categories, this scoping review identified key gaps in the social and behavioural evidence-base for HIV-serodiscordant couples. Designing studies that will contribute to evidence in these areas should inform future research. The social and behavioural aspects of biomedical prophylaxis (e.g., adherence to TasP or PrEP), and impacts on relationship quality and satisfaction were generally underexplored. Moreover, although intimate couple dynamics and emotional needs were important for understanding patterns of risk in qualitative studies, these factors were seldom addressed in quantitative work [38,54,64,66]. Negative impacts on relationship quality may affect the uptake of a range of risk management strategies including negotiated safety agreements, TasP, or PrEP [90,92-94,102,103]. In terms of prophylactic ART, studies that investigate associations between these strategies and relationship quality will be needed to better understand the impact on primary relationships over the long-term, especially at present when guidelines are shifting towards recommending these strategies. Relatedly, in studies that explored the effects of viral load perceptions on risk behaviours, actual or perceived viral load typically affected risk management choices [48-51,53]. For example, women who perceived ART to be protective reported increased sexual risk taking [104,105]. A systematic review addressing risk perceptions related to viral load and ART effectiveness would be useful.

There is a considerable amount of work that has investigated sexual risk behaviours or management of sexual risk, without taking account of the socio-structural factors that affect these outcomes. The social determinants of health are “core social processes and arrangements – reflective of social and cultural norms, values, networks, structures and institutions – that operate in concert with individual behaviours and practices to influence HIV epidemics in particular settings” [106], p. S294-5. For example, very few studies have explored the impact of criminalization of non-disclosure of HIV status on relationship quality [107]. The application of conceptual frameworks that take into account the effects of social and structural environments would facilitate a better understanding of the interplay of individual, dyadic, and social experience on relationship satisfaction within serodiscordant couples.

The only quantitative study focused on HIV-testing among HIV-negative partners was conducted in the Unites States prior to a change in national guidelines that recommended that higher risk individuals receive an annual HIV test. This study found that nearly three-quarters of HIV-negative partners in same-sex serodiscordant relationships had tested for HIV in the previous year [59,108,109]. Barriers or facilitators of HIV testing, especially for CHTC, were not specifically examined in any study focused on serodiscordant couples. CHTC is normally considered when the status of both partners is negative and/or unknown; however, studies that investigate the risks and benefits of involving the HIV-positive partner in the seronegative partner’s testing might inform the use of CHTC within this key population [110]. Since testing is crucial for prevention, understanding barriers to testing among HIV-negative partners and designing interventions to increase uptake should be a focus of future work.

Highly vulnerable non-IDU populations, including transgender persons and migrants, have been inadequately studied [45,46,87,97,111,112]. African-American people in the United States were represented within the series of papers generated by the *Eban* study that assessed the impact of a behavioural intervention on HIV risk behaviours within multiple urban settings in the United States [20,21]. The high infection rates observed among some African-American MSM may be attributable to low rates of diagnosis and treatment rather than patterns of risk behaviour [113], underscoring the importance of exploring research approaches that are sensitized to the social determinants of health.

Studies of reproductive issues among same-sex serodiscordant couples were entirely absent, despite the fact that 19% of same-sex couples in the United States were estimated to have children as of 2011 [114]. Although same-sex male couples do not experience transmission risk associated with procreative sex, the decision to have children and its impacts on relationship dynamics within this relationship context should be taken up in future studies.

Currently, very few studies have investigated barriers to disclosing a serodiscordant relationship to family and social networks, and the impacts of these decisions on the relationship. These impacts could be psychosocial, or may affect access to supportive services. In one of the very few studies on the subject, better perceived social integration facilitated a better likelihood of relationship disclosure by HIV-positive partners to their social networks when compared with their seronegative partners [88]. Given the tremendous stigma associated with HIV in particular settings, there is a need to better understand the impact of disclosure to family and social networks on access to supportive services and uptake of HIV risk management strategies within the couple. There were also very few studies that investigated associations between serostatus disclosure and sexual risk with primary partners. Future dyadic work would need to be limited to studies that aimed to recruit only the HIV-positive partner, given the couple’s self-identification as serodiscordant would need to be known by both the HIV-positive and HIV-negative partner in advance of recruitment. In cases where disclosure had not yet occurred between partners, it would not be possible to confirm the HIV-serodiscordant, primary relationship status of the couple that would be necessary for eligibility in a study that focused on this unit of analysis.

Only one-third of included studies recruited dyads, defined as both partners within a serodiscordant relationship. Therefore, the majority of studies relied on the responses of a single partner. Given the importance of interpersonal dynamics within a relationship, studies that recruit both partners of a serodiscordant couple are crucial for understanding the management of risk, the cultivation of intimacy within the couple, and changes over time. Dyadic studies also enhance the validity of outcome variables by devising ways of operationalizing the responses of both partners through techniques such as agreement scores. If couples-based approaches are to be employed within HIV prevention interventions, more studies focused on the dyad as the unit of analysis will be needed. Given the challenges of recruiting representative cohorts of HIV-serodiscordant dyads, methodological work that describes challenges and solutions would be helpful.

Population-based cohort studies would also help to increase our understanding of the epidemiological profile of serodiscordant couples, and would help to develop targeted programs that deliver single or combination HIV prevention interventions to couples that are most in need. Given the preponderance of studies focused on sexual risk behaviour and the movement away from viewing all unprotected anal or vaginal intercourse, or sex without condoms, as risky (i.e., due to variable risk associated with condomless sex across different risk environments), a review of the ways in which the evidence base has typically operationalized “risky sexual behaviour” would be a strong contribution to the literature. Finally, a major weakness of the literature was in the aggregation of results in studies that recruited mixed populations of couples (e.g., casual, primary, serodiscordant, seroconcordant) without presenting stratified results. This aggregation complicated comparability and synthesis of data. Therefore, if primary couples are to be considered different with respect to risk, they should be properly distinguished and discussed within larger studies.

Only eight intervention studies met the inclusion criteria for this scoping review. Published trials included behavioural interventions to reduce sexual risk [20-24], a psycho-educational group intervention to reduce depression and anxiety and increase marital satisfaction [25], an intervention to improve adherence to ART [26], and two interventions for improving social support [27,28]. Therefore, major gaps exists in relation to evaluated interventions designed to support serodiscordant couples in establishing a routine HIV-testing schedule, improving the quality of their relationships, supporting the navigation of negotiated safety agreements, and consistently executing seroadaptive strategies.

There were strengths and limitations to this review. Strengths included the thorough database searching and broad inclusion criteria (i.e., any study with a proportion of primary serodiscordant couples). Limitations included the omission of grey literature and reports published in languages other than English. Work that includes mixed cohorts, such as serodiscordant and seroconcordant couples, or primary and casual relationships, should identify these sample proportions clearly, provide consistent definitions, and conduct stratified analyses. Consistently defined outcomes and populations will facilitate helpful meta-analyses, the highest form of evidence.

## Conclusions

In summary, our findings highlight where future research on HIV-serodiscordant relationships may be directed, including the effects of risk management strategies on relationship quality, the social determinants of health for serodiscordant couples, dynamics around HIV testing, reproductive issues among same-sex couples, disclosure of serodiscordant status to social networks, dyadic studies, population-based studies and studies among vulnerable populations. Importantly, as HIV-positive partners are often the link to services and research, innovative ways are needed to reach out to HIV-negative partners in the context of research studies and supportive services. Overall, filling these gaps will be important for improving the health and quality of life for HIV-positive and HIV-negative partners engaged in serodiscordant relationships.

## Additional file

Additional file 1: Table S1. Sample systematic review search strategy used in MEDLINE*.
