# Supplementary material for: A scoping review and thematic analysis of social and behavioural research among HIV-serodiscordant couples in high-income settings
Source: BMC Public Health. 2015 Mar 13;15:241. doi: 10.1186/s12889-015-1488-9 (PMC4365541; doi:10.1186/s12889-015-1488-9)
Supplement: Additional file 1: Table S1. — Sample systematic review search strategy used in MEDLINE*. [file 12889_2015_1488_MOESM1_ESM.doc]

Additional file: Table S1: Sample systematic review search strategy used in MEDLINE*

| Step | Key word(s) | MeSH term(s) (where applicable) |
| --- | --- | --- |
| 1 | human immunodeficiency virus | human immunodefiency virus |
| 2 | HIV |  |
| 3 | acquired immune deficiency syndrome | acquired immunodefiency syndrome |
| 4 | AIDS |  |
| 5 | 1 OR 2 OR 3 OR 4 |  |
| 6 | relation* |  |
| 7 | couple* | couple |
| 8 | marriage* |  |
| 9 | partner* |  |
| 10 | spouse* |  |
| 11 | 6 OR 7 OR 8 OR 9 OR 10 |  |
| 12 | serodiscord* |  |
| 13 | 5 AND 11 AND 12 |  |
| *Similar strategies were used in other databases | | |
